# The response of cecal microbiota to inflammatory state induced by *Salmonella enterica* serovar Enteritidis

**DOI:** 10.3389/fmicb.2022.963678

**Published:** 2022-08-25

**Authors:** Geng Hu, Liying Liu, Xiuxiu Miao, Yanan Zhao, Yanan Peng, Lewen Liu, Xianyao Li

**Affiliations:** ^1^College of Animal Science and Technology, Shandong Provincial Key Laboratory of Animal Biotechnology and Disease Control and Prevention, Shandong Agricultural University, Tai'an, Shandong, China; ^2^College of Life Sciences, Shandong Agricultural University, Tai'an, Shandong, China

**Keywords:** chicken, *Salmonella*, inflammatory state, transcriptome, microbiome

## Abstract

By combining the experiments of reciprocal crosses of chicken infected with *Salmonella enterica* serovar Enteritidis (*S*. Enteritidis), we focused on the common response of cecal microbiota to an inflammatory state in respect of transcriptome and microbiome. The inoculation of *S*. Enteritidis improved the microbial diversity and promoted the microbiota evolution in our infection model. Correlation analysis between bacteria and inflammation-related genes showed that some intestinal microorganisms were “inflammophile” and thrived in an inflamed environment. The global function of cecal microbiome was to maintain the homeostasis likely by the up-regulation of microbial metabolism pathway in bacitracin, putrescine, and flavonoids production, although the bacitracin may affect the symbiotic bacteria Enterococcus. The action of *S.* Enteritidis had close relationships with multiple inflammation-related genes, including the genes *PTAFR*, *LY96*, and *ACOD1* which proteins are related to the binding and tolerance of LPS, and the genes *IL-18, IL-18R1* and *IL-18RAP* which products can form a functional complex and transmit IL-18 pro-inflammatory signal. Additionally, the infection of *S.* Enteritidis aroused the transcription of *EXFABP*, which protein has a potential to sequestrate the siderophore and might cause the decline of Escherichia-Shigella and Enterococcus. *S.* Enteritidis can escape from the sequestrating through the salmochelin, another kind of siderophore which cannot be recognized by EXFABP. Probably by this way, *S.* Enteritidis competed with the symbiotic bacteria and edged out the niches. Our research can help to understand the interplay between host, pathogen, and symbiotic bacteria.

## Introduction

Animal gastrointestinal tracts are highly populated with a diverse array of microorganisms that share a symbiotic relationship with their hosts and contribute to the overall health and disease state of the intestinal tract (Mon et al., 2015). Microbiota is so important even like an essential “organ,” yet this ecosystem remains incompletely characterized (Hooper and Gordon, 2001; Eckburg et al., 2005), especially when facing the invasion of pathogenic bacteria.

*Salmonella enterica* is a kind of facultative intracellular pathogens (Barrow, 1996; Kaiser et al., 2000). One of its serotypes is *Salmonella enterica* serovar Enteritidis (*S*. Enteritidis), which is most frequently associated with diarrheal disease in humans but can colonize chicken intestine with little clinical symptoms (Awad et al., 2012). In chickens, the immune response to *S*. Enteritidis infection switches from earlier pro-inflammatory to later anti-inflammatory tolerance within a few days. Microorganisms in the gut undergo selective pressure from the host as well as from microbial competitors (Arumugam et al., 2011). This situation is more stern for invasive *Salmonella*, which has evolved multiple mechanisms to outgrow other bacteria in hostile environment of the inflamed gut, including tetrathionate respiration (Winter et al., 2010), nitrate respiration (Lopez et al., 2012), ethanolamine nutrition (Thiennimitr et al., 2011), salmochelin siderophore (Raffatellu et al., 2009), and the triggered inflammation itself.

Modest inflammation can help the host to resist the invasion of pathogens and is favored by partial microorganisms. Once excessively, it will damage the health of host and the normal community of microbiota. Understandably, the commensal bacteria can actively coordinate the immune tolerogenic response of host (Fava and Danese, 2011). We herein examine the chickens infected with *S*. Enteritidis in respect of transcriptome and microbiome for the interplay between host, pathogen, and symbiotic bacteria, and further knowledge of this microcosm.

## Materials and methods

### Animals and experimental design

As described previously (Hu et al., 2022), the chicks used in experiment were two populations of reciprocal crosses [the Cross (Guangxi Yao ♂ × Jining Bairi ♀) and the Reverse-cross (Guangxi Yao ♀ × Jining Bairi ♂)], which derived from two Chinese local chickens, Guangxi Yao and Jining Bairi. For each crossbreed, 100 mixed gender, *Salmonella*-negative birds were randomly divided into two groups and housed in separated isolators (32 to 35°C, 50 to 60% RH, and 24 h light) with *ad libitum* access to water and antibiotic-free feed. The strain of *S*. Enteritidis (CVCC3377) was purchased from China Veterinary Culture Collection Center (Beijing, China). After rejuvenation with nutrient broth (Hopebio, Qingdao, China), the bacterial solution was centrifuged at 4,000 rpm for 5 min and suspended again with sterilized PBS to 1.0 × 10^8^ cfu/ml. At 2-day old, the birds of treatment group were orally inoculated with 0.3 ml *S*. Enteritidis, and the birds of control group were mock inoculated with the same volume of PBS. At 3 days post-inoculation (dpi), 9 to 12 birds in each group were sacrificed. The cecal contents and cecum tissues were collected aseptically for 16S rRNA gene sequencing and RNA sequencing, respectively. The experimental procedure was repeated on another crossbreed. Thus four groups were generated as Cross Control (CC, *n* = 3 for transcripomic sequencing and 11 for microbiomic sequencing), Cross Treatment (CT, *n* = 3 and 11), Reverse-cross Control (RC, *n* = 3 and 9), and Reverse-cross Treatment (RT, *n* = 3 and 9). The animal experiment was approved by the Laboratory Animal Management and Use Committee of Shandong Agricultural University (Permit Number: SDAUA-2018-058). We strove to reduce the suffering of animals.

As described in our previous study (Hu et al., 2022), to focus on the common characteristics across the two crossbreeds and increase the reliability of the analysis, we combined the experiments of reciprocal crosses. That is, the control groups (CC and RC) and the treatment groups (CT and RT) were merged into MC and MT, respectively. Thus, the sample size was 6 and 20 in each merged group for transcriptome and microbiome, respectively. The theoretical basis of this method is: (i) the circumstances and the operation were repeated to guarantee the experimental consistence; (ii) the chicken genotype had reported having a limited effect on resistance to *S*. Enteritidis infection between two inbred lines (Mon et al., 2015) and our reciprocal crosses were speculated to have a similar genetic resistance to pathogens; (iii) the alpha diversity estimates with Shannon (Supplementary Figure S1) and Simpson (Supplementary Figure S2) indexes were comparable between the reciprocal crosses of non-infected chickens.

### Total RNA isolation, library preparation and sequencing

The details were described in our previous study (Hu et al., 2022). RNA sequencing and the preliminary analysis were completed in Shanghai OE Biotech Co., Ltd. (Shanghai, China).

### Bacterial DNA extraction and 16S rRNA gene sequencing

Bacterial DNA was extracted from the samples of cecal contents using the OMEGA DNA Kit (Omega Bio-Tek, Norcross, United States) according to manufacturer’s protocols. The final DNA concentration and purification were determined by NanoDrop 2000 (Thermo Scientific, Wilmington, United States), and DNA quality was checked by 1% agarose gel electrophoresis. The V3-V4 region of the bacteria 16S rRNA gene was amplified by PCR using the following primer pair: 338F, ACTCCTACGGGAGGCAGCAG, and 806R, GGACTACHVGGGTWTCTAAT. PCR reactions were performed in 20 μl mixture containing 4 μl 5 × FastPfu Buffer, 2 μl 2.5 mM dNTPs, 0.8 μl 5 μM each primer, 0.4 μl FastPfu Polymerase and 10 ng template DNA under the following cycling conditions: 3 min at 95°C followed by 27 cycles of 30 s at 95°C, 30 s at 55°C and 45 s at 72°C, and a final 10 min at 72°C. The pooled products were paired-end sequenced (2 × 300) on the Illumina MiSeq platform (Illumina, San Diego, United States) according to standard protocols.

Raw fastq files were demultiplexed, quality-filtered by Trimmomatic and merged by FLASH with the following criteria: (i) The reads were truncated at any site receiving an average quality score < 20 over a 50 bp sliding window. (ii) Primers were exactly matched allowing 2 nucleotide mismatching, and reads containing ambiguous bases were removed. (iii) Sequences whose overlap longer than 10 bp were merged according to their overlap sequence. Operational taxonomic units (OTUs) were clustered with 99% similarity cut-off using UPARSE and chimeric sequences were identified and removed using UCHIME. The taxonomy of each 16S rRNA gene sequence was analyzed by RDP Classifier algorithm against the Silva138 16S rRNA database using confidence threshold of 70%. 16S rRNA gene sequencing and the preliminary analysis were completed in Majorbio Biotech Co., Ltd. (Shanghai, China).

### Statistical analysis

We performed the analyses of DESeq2, BugBase, PICRUSt1, Wilcoxon rank-sum test, Studen’s *t* test and so on, which details were recorded in the results. Most of them were run on the OEBiotech Cloud^1^ and the Majorbio Cloud.^2^

### Availability of data

The transcriptome data and the microbiome data were deposited in National Genomics Data Center^3^ under BioProject accession number CRA005292 and CRA004605, respectively.

## Results

### The triggered inflammatory response in cecum tissue

The result of 16S rRNA gene sequencing showed that *S*. Enteritidis (OTU607) was detected in treatment groups (CT and RT) but not in the control groups (CC and RC; Supplementary Table S1), suggesting the success of challenge work. As described in our previous study (Hu et al., 2022), after the merging, there were total 15,205 genes in RNA-seq dataset of 12 samples and 525 differentially expressed genes (|log_2_FC| > 1 and *Padj* < 0.05; Supplementary Table S2), of which 394 and 131 were up-and down-regulated compared to the control (MC), respectively. The RNA-seq data were validated with 29 genes by RT-qPCR. All the 30 genes enriched in inflammatory response term were up-regulated significantly and the NF-κB signaling pathway was activated at mRNA level. Importantly, the negative regulators of NF-κB signal, ACOD1 and TNIP3, and the anti-inflammatory cytokine IL-10 were all increased sharply at mRNA level. The above data indicated that the innate immune response was triggered and was transforming to the anti-inflammatory tolerance at 3 dpi.

### Microbial richness and diversity

16S rRNA gene sequencing of 40 samples generated 2,386,945 high-quality sequence reads (415 nt in average), which were clustered into 943 OTUs (Supplementary Table S1) including 10 phyla, 15 classes, 39 orders, 66 families, 122 genera, and 198 species. All the samples were rarefied to 30,221 reads. The Firmicutes phylum had the most OTUs (798, 84.6%) and reads (864,800, 71.5%), following Proteobacteria (86, 9.1%; 341,097, 28.2%), Bacteroidota (28, 3.0%; 189, 0.02%), Actinobacteriota (11, 1.2%; 1,469, 0.12%), Patescibacteria (1, 0.1%; 2, ~0%), Verrucomicrobiota (1, 0.1%; 2, ~0%), Acidobacteriota (1, 0.1%; 1, ~0%), Elusimicrobiota (1, 0.1%; 1, ~0%), Synergistota (1, 0.1%; 1, ~0%), and some unclassified OTUs (15, 1.6%; 1,278, 0.11%).

**Figure 1:**
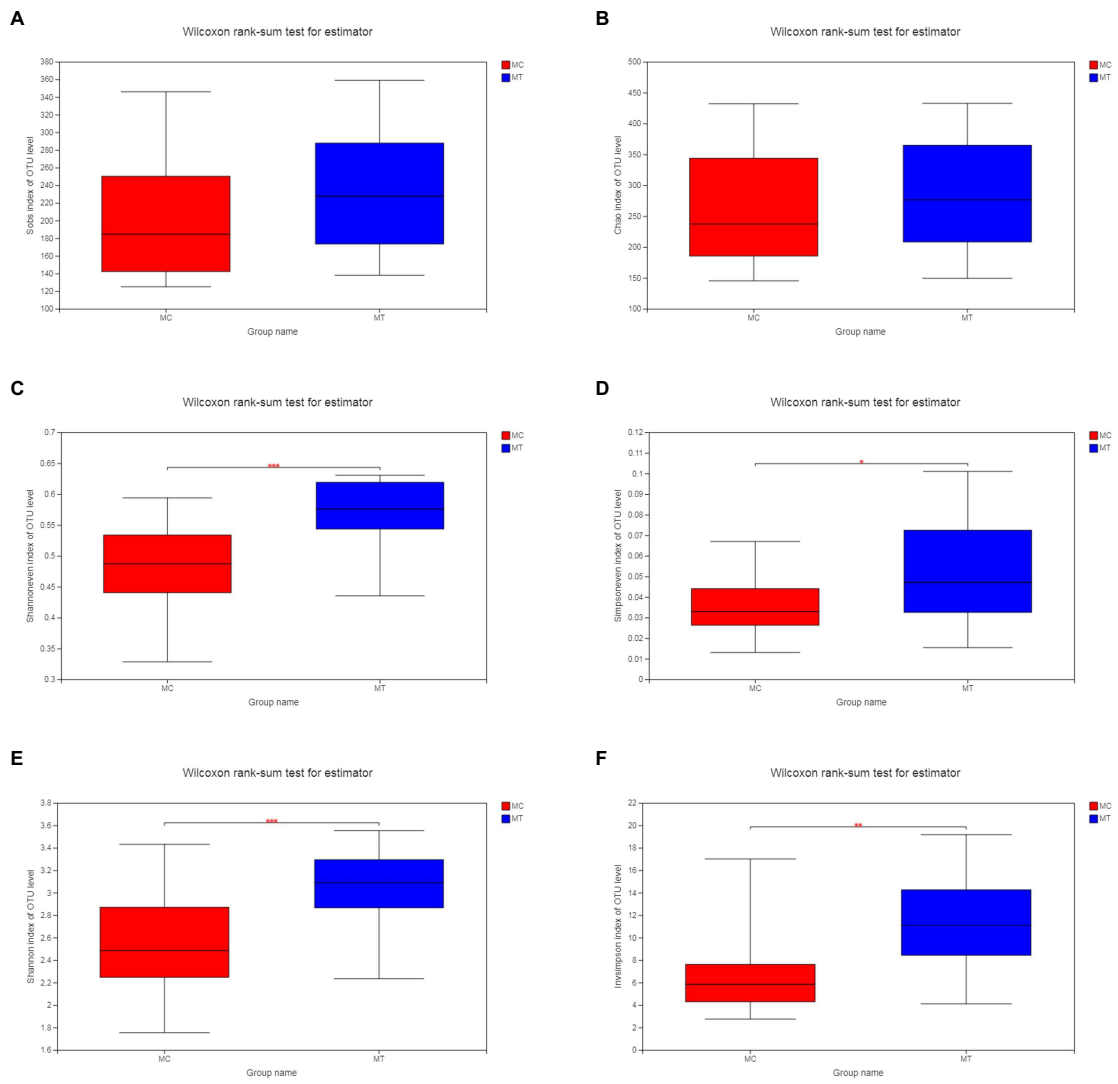
Alpha diversity of cecal microbiota. **(A)** Sobs index; **(B)** Chao index; **(C)** Shannoneven index; **(D)** Simpsoneven index; **(E)** Shannon index; and **(F)** Invsimpson index.

As described in Materials and Methods, we merged the control groups (CC and RC) and the treatment groups (CT and RT) into MC (*n* = 20) and MT (*n* = 20), respectively. Analysis on OTU level showed that the richness estimate with Sobs (Figure 1A) and Chao (Figure 1B) indexes was comparable between MC and MT (*p* = 0.105 and 0.365, respectively), but the latter had a tendency of increase. At the same time, the evenness estimate with Shannoneven (Figure 1C) and Simpsoneven (Figure 1D) indexes was increased significantly after inoculation (*p* = 4.2E-04 and 0.029, respectively). Under the co-action of richness and evenness, the diversity estimate of MT with Shannon (Figure 1E) and Simpson (Figure 1F) indexes had a marked increase compared to MC (*p* = 8.4E-04 and 0.002, respectively). It should be noted that the diversity analysis within each crossbreed had the similar result (Supplementary Figures S1, S2).

### Microbial community composition

**Figure 2:**
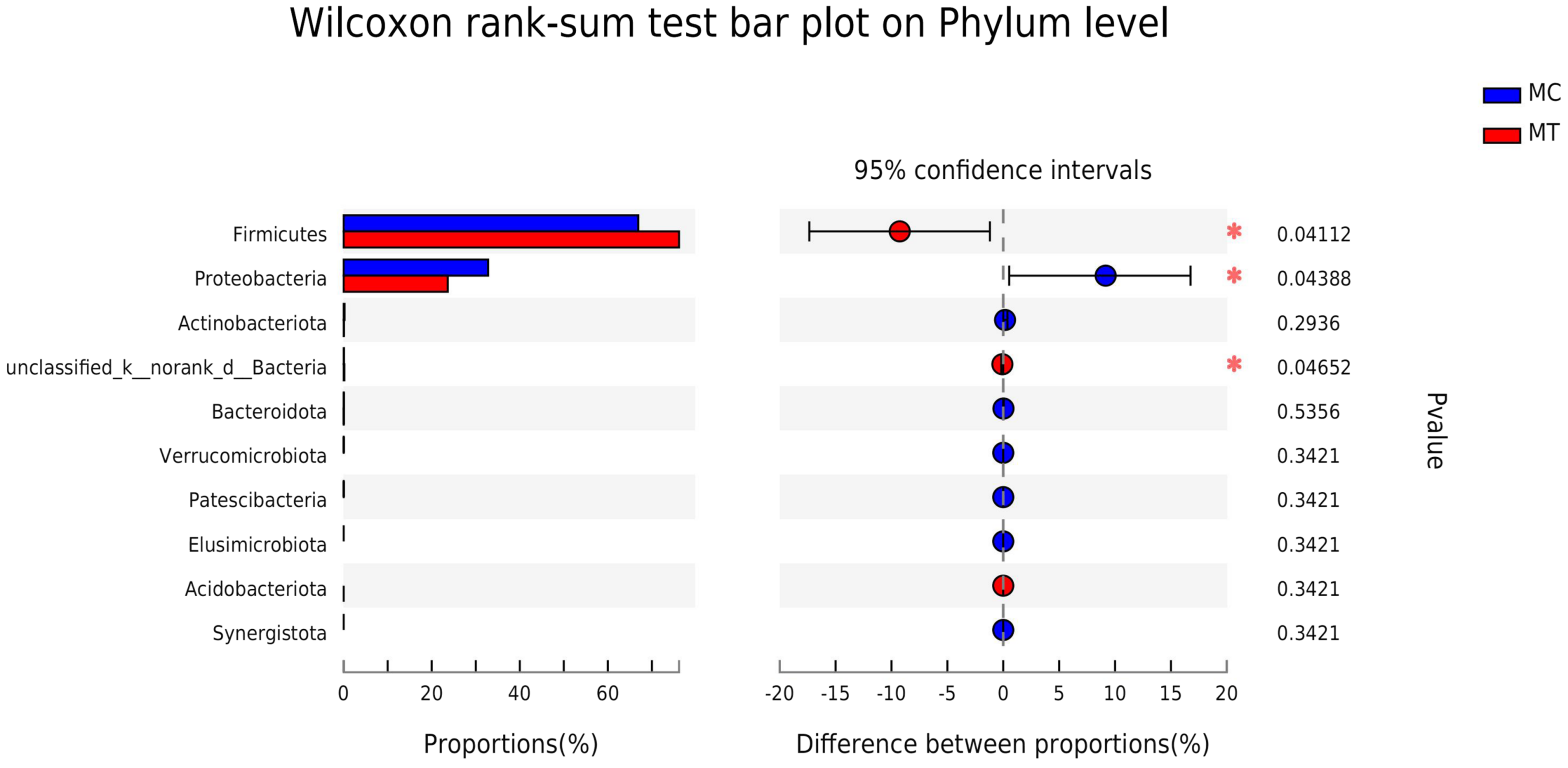
Difference analysis on Phylum level.

The Firmicutes and Proteobacteria phyla were dominant in the cecal microbiota of 5-day-old chickens regardless of whether *S*. Enteritidis was inoculated or not (Figure 2). Compared to control, Firmicutes increased from 66.91 to 76.17% (*p* = 0.041) while Proteobacteria decreased from 32.80 to 23.64% (*p* = 0.044).

**Figure 3:**
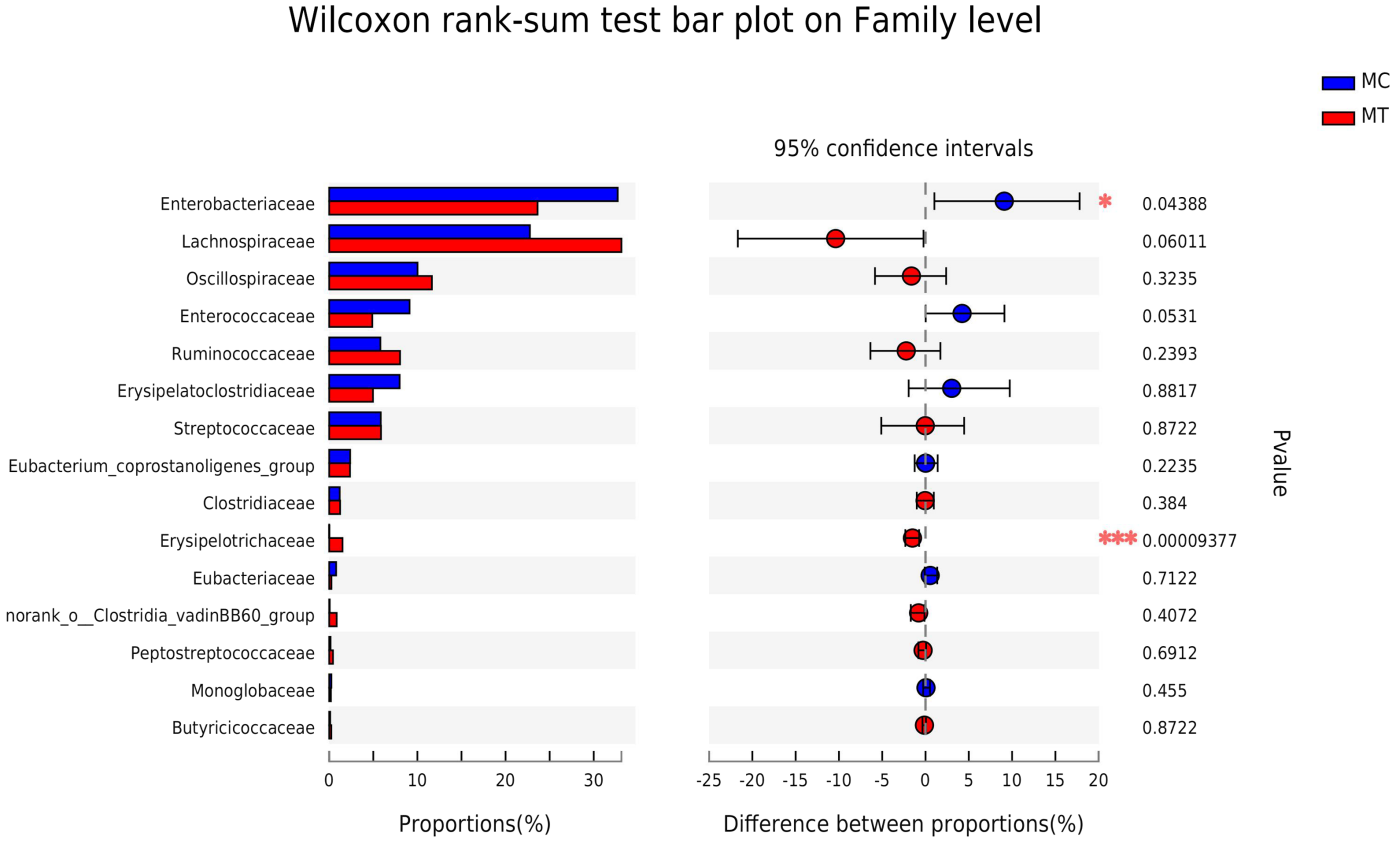
Difference analysis on Family level.

**Figure 4:**
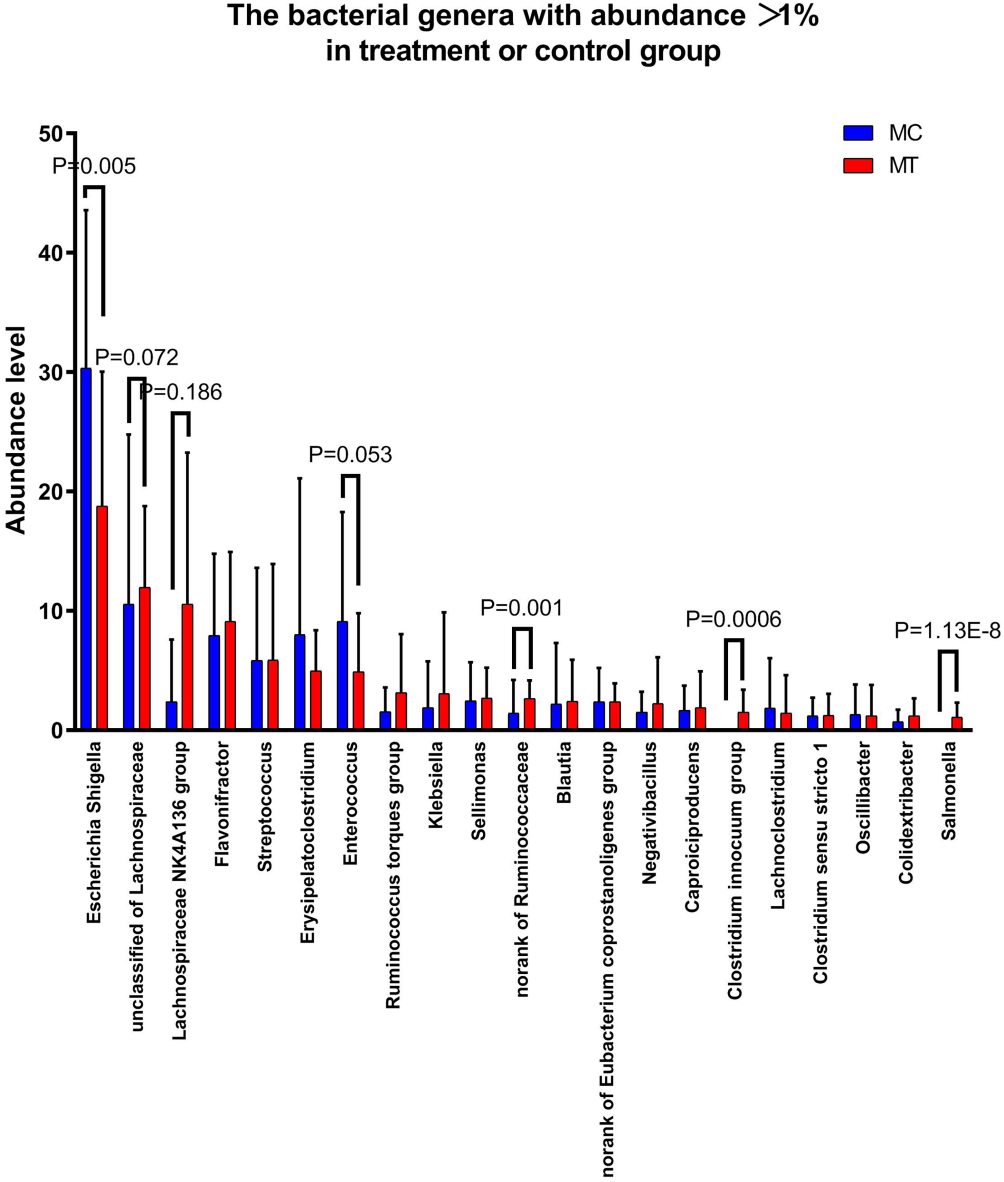
The top 21 genera with abundance >1% in treatment or control group.

Further analysis on family (Figure 3) and genus (Figure 4) levels showed that the decline of Proteobacteria phylum was principally caused by Enterobacteriaceae family (from 32.72 to 23.63%, *p* = 0.044) and its Escherichia-Shigella genus (from 30.32 to 18.75%, *p* = 0.005), though its two other genera Salmonella (from 0 to 1.09%, *p* = 1.13E-08) and Klebsiella (from 1.87 to 3.07%, *p* = 0.304) had an increase compared to control.

For Firmicutes phylum, the Lachnospiraceae family had the largest increase after *S*. Enteritidis inoculation (from 22.76 to 33.14%, *p* = 0.060; Figure 3), which was mainly attributed to its two genera, an unclassified (from 10.56 to 11.95%, *p* = 0.072) and NK4A136 group (from 2.38 to 10.54%, *p* = 0.186; Figure 4). The secondary contribution to the increase of Lachnospiraceae family was from Ruminoccus torques group (from 1.53 to 3.13%, *p* = 0.507), Sellimonas (from 2.45 to 2.70%, *p* = 0.218) and Blautia (from 2.19 to 2.40%, *p* = 0.377). A norank genus of Ruminococcaceae family was increased significantly (from 1.42 to 2.64%, *p* = 0.001) though the overall increase of this family was not marked (*p* = 0.239). Erysipelotrichaceae family was almost composed of Clostridium innocuum group, which increased markedly after inoculation (from ~0 to 1.49%, *p* = 5.9E-04). It is worth noting that, contrary to the overall up-regulation of Firmicutes phylum, Enterococcaceae family and its only Enterococcus genus decreased notably after inoculation (from 9.11 to 4.89%, *p* = 0.053).

### The predicted phenotypes and functions of cecal microbiota

**Figure 5:**
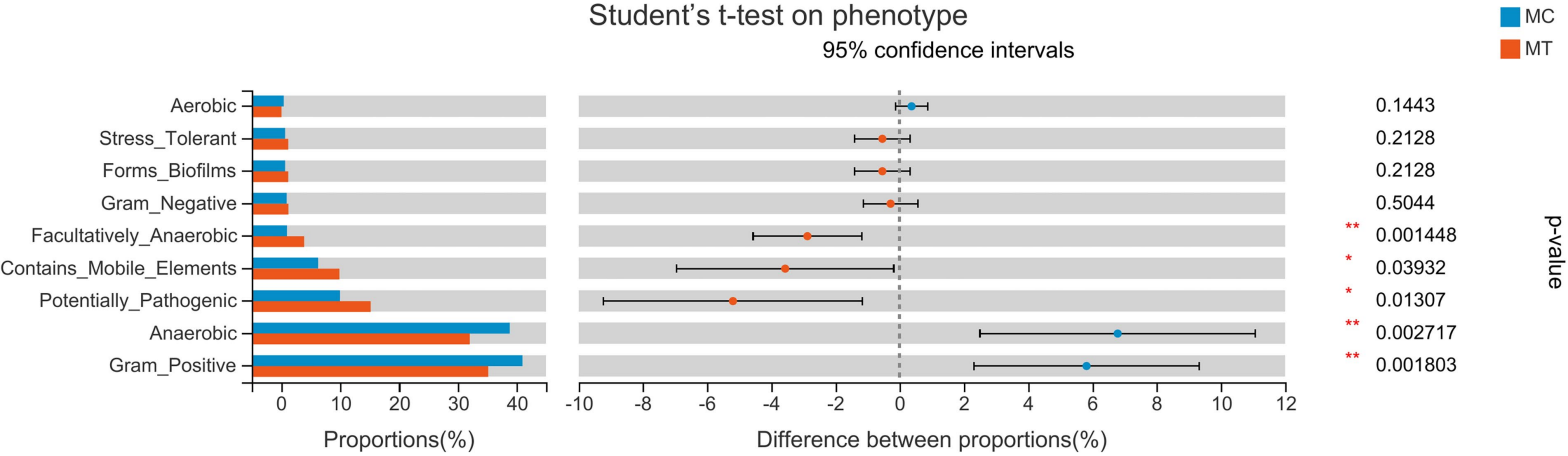
The predicted phenotypes.

To explore the influence of microbiota change on phenotype, we used the BugBase tool to predict the phenotypes (Figure 5). After inoculation, the Gram-positive bacteria and the anaerobes were decreased (*p* < 0.05), but the facultative anaerobes, the bacteria containing mobile elements, and the potential pathogen were increased (*p* < 0.05). As *S*. Enteritidis only contributed about 1% of the proportion, we deduced that the phenotypic differences were derived from cecal microbiota rather than *S*. Enteritidis alone.

In addition, we used the PICRUSt1 tool to predict the functions of cecal microbiota. Among the 125 metabolic pathways on level three (Supplementary Table S3), 9 pathways were screened out (1.3 foldchanges between MC and MT, *p* < 0.05, Student’s *t* test), including D-Arginine and D-ornithine metabolism (FC = 3.07, *p* = 8.5E-05), Flavonoid biosynthesis (FC = 2.80, *p* = 0.001), Flavone and flavonol biosynthesis (FC = 2.70, *p* = 0.006), Steroid hormone biosynthesis (FC = 2.59, *p* = 0.007), N-Glycan biosynthesis (FC = 2.52, *p* = 0.003), Atrazine degradation (FC = 1.88, *p* = 0.006), Other glycan degradation (FC = 1.64, *p* = 0.023), Biosynthesis of siderophore group nonribosomal peptides (FC = 0.72, *p* = 0.027), and Fluorobenzoate degradation (FC = 0.67, *p* = 0.023).

### The relationship between bacteria and the inflammation-related genes

**Figure 6:**
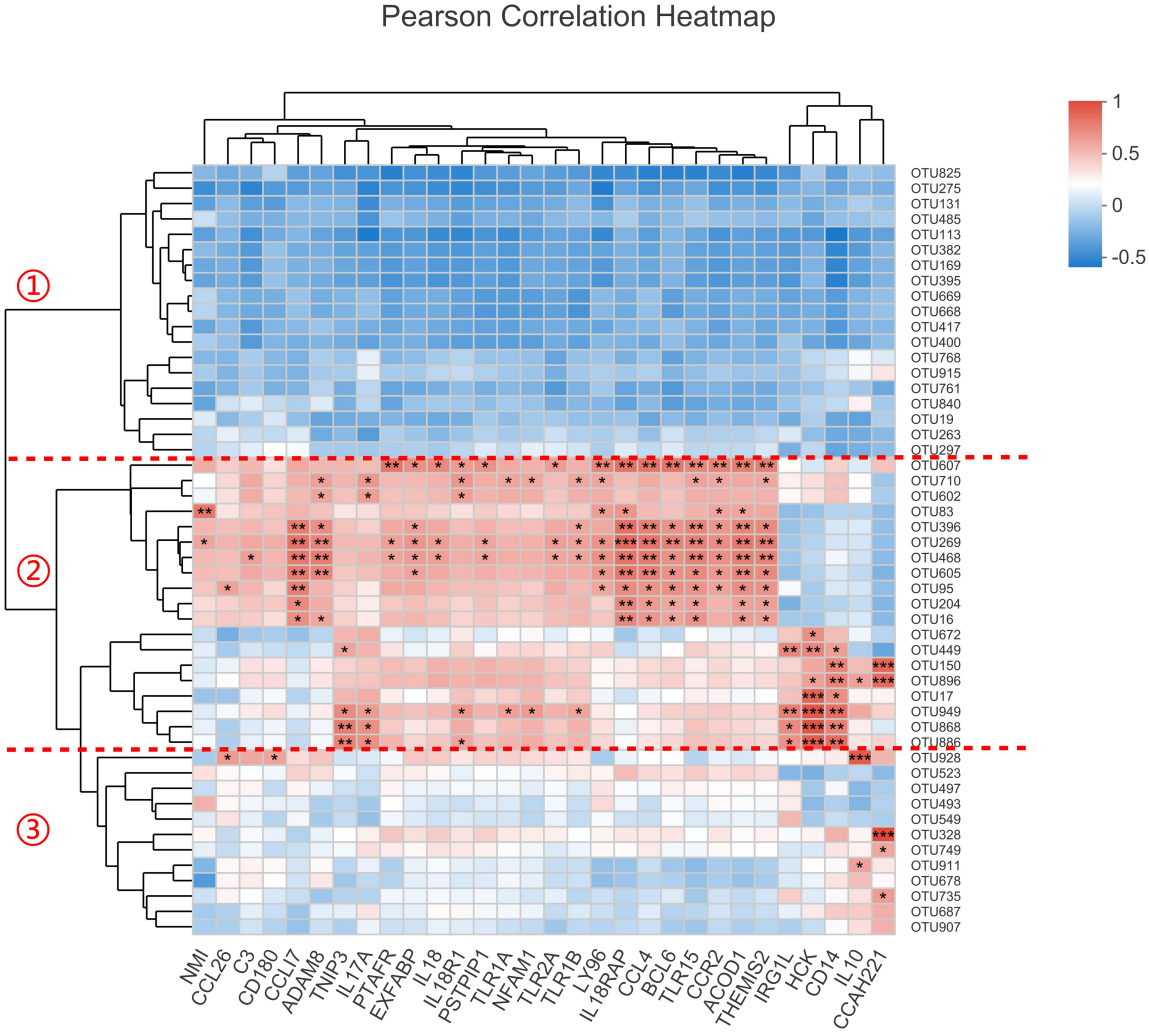
The correlation analysis between the top50 OTUs and 30 inflammation-related genes. The significant level in figure is marked as *(0.01 ≤ *p* < 0.05), **(0.001 ≤ *p* < 0.01), and ***(*p* < 0.001).

The host’s genes can not affect intestinal bacteria directly, but there must be some indirect relationships between them, especially for the inflammation-related genes. As the 12 transcriptomic samples were overlapped with the microbiomic samples, we constructed the correlation analysis with the most abundant 50 OTUs and the 30 genes enriched in inflammatory response term (Figure 6). The result showed that these OTUs were obviously divided into three categories. Category 1 is generally negatively correlated with these genes while category 2 is positively correlated with most of them. Relatively, category 3 has little relationship with these genes. It was known that all the 30 inflammation-related genes were up-regulated after inoculation. We deduced that the OTUs in category 1 should be decreased and the OTUs in category 2 be increased. Consistantly, after examining our 16S rRNA gene sequencing data, we found that all the OTUs with *p* < 0.1 (Student’s *t*-test between MC and MT) in category 1 were decreased after inoculation, including OTU825 (Escherichia-Shigella), OTU275 (Enterococcus), OTU669 (an unclassified of Lachnospiraceae), and OTU668 (an unclassified of Lachnospiraceae), while all the OTUs with *p* < 0.1 in category 2 were increased after inoculation, including OTU607 (Salmonella), OTU269 (Negativibacillus), OTU150 (Flavonifractor), OTU896 (Clostridium innocuum group), OTU949 (a norank of Ruminococcaceae), OTU868 (an unclassified of Lachnospiraceae), and OTU886 (an unclassified of Lachnospiraceae). We inferred that the microbes in category 2 were more adaptable to the inflamed cecal environment than the ones in category 1.

Additionally, we found *S*. Enteritidis (OTU607) was correlated positively with genes *PTAFR, EXFABP, IL18, IL18R1, IL18RAP, PSTPIP1, TLR2A, LY96, CCL4, BCL6, TLR15, CCR2, ACOD1*, and *THEMIS2* (*p* < 0.05). By retriving the functions of these genes in NCBI, we found that the proteins encoded by *PTAFR, LY96,* and *ACOD1* are related to the binding and tolerance of lipopolysaccyaride (LPS); the products of *IL-18, IL-18R1* and *IL-18RAP* can form a functional complex and transmit IL-18 pro-inflammatory signal, which is capable of stimulating interferon gamma production [the gene *IFNG* was increased by 18.06 times (*Padj* = 3.33E-06) at mRNA level in our RNA-seq data], and of regulating both T helper (Th) 1 and Th2 responses. Importantly, *EXFABP* [increased by 14.19 times (*Padj* = 5.43E-09) in our RNA-seq data and 14.62 times (*p* = 0.006, Student’s t test) in RT-qPCR data compared to control] encodes a conserved domain (NCBI: cd19439), which is bacteriostatic and tightly binds the 2,3-catechol-type ferric siderophores associated with enteric bacteria and Gram-positive bacilli. These data indicated that the host’s inflammatory response was closely related to the *S*. Enteritidis infection.

## Discussion

Bacterial colonization of the intestinal tract begins after birth or hatching, or earlier (Ding et al., 2017; Lee et al., 2019). In chickens, initial gut colonizers are recruited from facultative anaerobes of the Enterobacteriaceae family (Proteobacteria phylum), following the representatives of Firmicutes within a week after hatching and the representatives of Bacteroidetes lastly (Videnska et al., 2014). According to the clustering result, Videnska et al. (2014) defined four developmental stages of microbiota composition during the chicken’s whole life (60 weeks of age). In the first stage (the first week), the cecal microbiota was dominated by Proteobacteria and Firmicutes phyla (~99.9% of abundance), which is consistent with our data. Importantly, the primary and secondary roles of the two phyla were reversed on day 4 and 7 in their study. The Firmicutes increased from 34.4 ± 17.9% to 66.5 ± 7.31% while the Proteobacteria decreased from 65.5 ± 17.9% to 33.3 ± 7.30%. At the same time, both the observed OTU numbers and the chao1 index were increased markedly, reflecting the increase of microbiota richness. The tendencies of change on phyla and richness in their study are coherent to our result of *S*. Enteritidis treatment compared to control. It is suggested that the inoculation of *S*. Enteritidis, a non-host specific pathogen, can phenotypically promote the evolution of chicken microbiota from single species to a complex community at newly hatching period. This might be due to the inflammation and the environmental changes, which broke the original ecological balance and induced some low-abundance species to proliferate.

Our result at this point is contrary to previous studies (Mon et al., 2015; Liu et al., 2018), which reported the diversity of cecal microbiota was reduced after *S*. Enteritidis inoculation. This discrepancy may be caused by many factors including bacterial strains and concentration, chicken breeds and age, etc. For example, Mon et al. (2015) inoculated the chicks at 1-day old and sampled at 2 and 7 dpi, while we did them at 2-day old and 3 dpi, respectively. As well known, the bacterial community succeeds rapidly and may exhibit radical shifts at a daily scale (Gilbert et al., 2010; Cong et al., 2017), especially in the first week after hatching.

Function prediction of cecal microbiome by PICRUSt1 found nine metabolic pathways changed significantly compared to control. It is worth noting that D-Arginine and D-ornithine metabolism is a module of D-Amino acid metabolism (KEGG: ko00470). There are two key products derived from this module, bacitracin and putrescine. Bacitracin is a natural polypeptide antibiotic and interferes with the peptidoglycan synthesis of Gram-positive microorganisms (Butaye et al., 2003). Putrescine has been demonstrated to promote animal growth and facilitate the recovery of intestinal epithelium after infection (Girdhar et al., 2006). They might contribute to the decrease of Gram-positive Enterococcus genus and the restitution of infected epithelium, respectively. The other two pathways Flavonoid biosynthesis (KEGG: ko00941) and Flavone and flavonol biosynthesis (KEGG: ko00944) can synthesize a variety of flavonoids, which exhibit diverse biological properties such as anti-inflammatory (Feng et al., 2012), antimicrobial (Huang et al., 2014), and cytotoxic (He et al., 2022). Flavonoids might be employed by microbiota as a kind of colonization resistance to invasive *S*. Enteritidis. Another important metabolic pathway is the Biosynthesis of siderophore group (KEGG: ko01053). Some bacteria including *Escherichia coli* (Julien et al., 2020) possess this pathway and produce siderophores to promote the uptake of iron, which is of great significance in their colonization, growth, and invasion. This pathway’s reduction should be associated with a significant decrease in Escherichia-Shigella genus. Based on the above information, we deduced that the cecal microbiome might exert colonization resistance and help to maintain homeostasis by producing the bacitracin, putrescine, and flavonoids. However, the bacitracin can affect the symbiotic Gram-positive Enterococcus, which maybe caused the decline of this genus.

Microorganisms have evolved the ability to thrive in host gastrointestinal tract, including in states of inflammation (Mukhopadhya et al., 2012). According to the result of the correlation analysis (Figure 6), we defined three categories of bacteria. The bacteria of category 1, represented by genera Escherichia-Shigella and Enterococcus, were sensitive to inflammtory factors and reduced in this state. The bacteria of category 2, represented by genera Salmonella, Negativibacillus, Flavonifractor, and Clostridium innocuum group, could thrive in the inflamed environment. These bacteria may be the so-called “inflammophile” organisms, which in turn can perpetuate the inflammation to support their own survival (Mukhopadhya et al., 2012). It should be noted that there were two unclassified genera of Lachnospiraceae in each of these two categories (OTU668 and OTU669 in category 1; OTU868 and OTU886 in category 2), indicating the functional diversity of this family.

In a study of obesity and diabetes in mice, Cani et al. (2008) evidenced that gut bacteria are involved in high-fat diet-induced metabolic endotoxemia and inflammation, which attributes to the increase of gut permeability and enhancement of LPS absorption. As well known, the intestinal epithelium acts as a barrier to avoid LPS translocation, but some endogenous or exogenous factors may alter this function, such as alcohol consumption and pathogen invasion. *Salmonella* can entry into epithelial cells *via* a type III secretion system, which is present and conserved in all *Salmonella* serotypes tested (Ochman and Groisman, 1996; Darwin and Miller, 1999). This action and the subsequent inflammation will inevitably cause intestinal epithelium and mucosa damage, leading to a leaky gut and ascended plasma LPS levels.

In accordance with this research, the genes *PTAFR, LY96,* and *ACOD1* were increased notably in our data, symbolizing the enhancement of the binding and tolerance of LPS. It was rational to infer that the invasion of *S*. Enteritidis gave rise to the increase of facultative anaerobes, potential pathogens, and Gram-negative bacteria (Figure 5), and a natural rise of LPS in cecal contents, which continuously entered into the system through the injured epithelium and sustain the immune response of the host.

The host’s EXFABP can tightly bind the ferric siderophores, which are associated with enteric bacteria and Gram-positive bacilli (NCBI: cd19439). The marked decrease of Enterobacteriaceae family and Enterococcaceae family (Bacilli class) let us image that *S*. Enteritidis aroused the inflammation and EXFABP protein to limit the growth of the two species, and competed for their nutrients and niches. At the same time, *S*. Enteritidis produced two siderophores, enterobactin and salmochelin. The latter is not recognized by EXFABP, thus allowing the *S*. Enteritidis to escape EXFABP-mediated growth inhibition under iron restriction (Julien et al., 2020). Additionally, Firmicutes is characterized by the acquisition of Fe^2+^ *via* the FeoAB transport system and is independent of the siderophore (Polansky et al., 2015; Flannagan et al., 2022), which may be one of the causes that led to its increase after *S*. Enteritidis inoculation.

## Conclusion

In an inflammatory state induced by *S.* Enteritidis, the response of cecal microbiota was diverse. Some bacteria such as Salmonella, Negativibacillus, Flavonifractor, and Clostridium innocuum group had a preference for inflammation and outgrew other bacteria. The action of *S.* Enteritidis had close relationships with multiple inflammation-related genes, such as *PTAFR*, *LY96*, *ACOD1, IL-18, IL-18R1* and *IL-18RAP*. Additionally, the challenge of *S.* Enteritidis aroused the transcription of *EXFABP*, which protein can sequestrate the siderophore secreted by enteric bacteria and Gram-positive bacilli, such as Escherichia-Shigella and Enterococcus. *S.* Enteritidis can escape from the sequestrating through the particular salmochelin, another kind of siderophore which cannot be recognized by EXFABP. We assumed that, probably by this way, *S.* Enteritidis competed with its relatives and other symbiotic bacteria, and profited from others’ misfortune.

## Data availability statement

The data presented in the study are deposited in National Genomics Data Center, accession number CRA005292 and CRA004605.

## Ethics statement

The animal study was reviewed and approved by Laboratory Animal Management and Use Committee of Shandong Agricultural University.

## Author contributions

LiL and XL planned and designed the research and experiments. GH, XM, YZ, and LeL undertook the animal trial and the processing of samples. GH and YP performed the experiments and analyzed the data. GH, LiL, and XL wrote the paper. All authors contributed to the article and approved the submitted version.

## Funding

This work was supported by National Natural Science Foundation of China (31601980 and 31872343), National Key Research and Development Program of China (2021YFD1300102), Shandong Modern Agricultural Industry and Technology System (SDAIT-11-02), and Shandong Provincial Natural Science Foundation (ZR2018MC026).

## Conflict of interest

The authors declare that the research was conducted in the absence of any commercial or financial relationships that could be construed as a potential conflict of interest.

## Publisher’s note

All claims expressed in this article are solely those of the authors and do not necessarily represent those of their affiliated organizations, or those of the publisher, the editors and the reviewers. Any product that may be evaluated in this article, or claim that may be made by its manufacturer, is not guaranteed or endorsed by the publisher.

## Footnotes

^1^ https://cloud.oebiotech.cn

^2^ www.majorbio.com

^3^ https://ngdc.cncb.ac.cn/

## Supplementary material

SUPPLEMENTARY FIGURE S1 Shannon index of CC, CT, RC, and RT.

SUPPLEMENTARY FIGURE S2 Simpson index of CC, CT, RC, and RT.

SUPPLEMENTARY TABLE S1 The 943 OTUs in 16S rRNA gene sequencing.

SUPPLEMENTARY TABLE S2 The 525 differentially expressed genes in RNA-seq.

SUPPLEMENTARY TABLE S3 The 125 metabolic pathways in PICRUSt1 prediction.

## References

[ref1] Arumugam M. Raes J. Pelletier E. Le Paslier D. Yamada T. Mende D. R.. (2011). Enterotypes of the human gut microbiome. Nature. 473, 174–180. doi: 10.1038/nature09944, PMID: 21508958 PMC3728647

[ref2] Awad W. A. Aschenbach J. R. Khayal B. Hess C. Hess M. (2012). Intestinal epithelial responses to salmonella enterica serovar Enteritidis: effects on intestinal permeability and ion transport. Poult. Sci. 91, 2949–2957. doi: 10.3382/ps.2012-02448, PMID: 23091155

[ref3] Barrow P. A. (1996). Immunity to salmonella and other bacteria. Poult. Immuno. Poult. Sci. Symposium Series. 24, 243–263.

[ref4] Butaye P. Devriese L. A. Haesebrouck F. (2003). Antimicrobial growth promoters used in animal feed: effects of less well known antibiotics on gram-positive bacteria. Clin. Microbiol. Rev. 16, 175–188. doi: 10.1128/CMR.16.2.175-188.2003, PMID: 12692092 PMC153145

[ref5] Cani P. D. Bibiloni R. Knauf C. Waget A. Neyrinck A. M. Delzenne N. M.. (2008). Changes in gut microbiota control metabolic endotoxemia-induced inflammation in high-fat diet-induced obesity and diabetes in mice. Diabetes. 57, 1470–1481. doi: 10.2337/db07-1403, PMID: 18305141

[ref6] Cong X. Judge M. Xu W. Diallo A. Janton S. Brownell E. A.. (2017). Influence of feeding type on gut microbiome development in hospitalized preterm infants. Nurs. Res. 66, 123–133. doi: 10.1097/NNR.0000000000000208, PMID: 28252573 PMC5334772

[ref7] Darwin K. H. Miller V. L. (1999). Molecular basis of the interaction of salmonella with the intestinal mucosa. Clin. Microbiol. Rev. 12, 405–428. doi: 10.1128/CMR.12.3.405, PMID: 10398673 PMC100246

[ref8] Ding J. Dai R. Yang L. He C. Xu K. Liu S.. (2017). Inheritance and establishment of gut microbiota in chickens. Front. Microbiol 8:1967. doi: 10.3389/fmicb.2017.01967, PMID: 29067020 PMC5641346

[ref9] Eckburg P. B. Bik E. M. Bernstein C. N. Purdom E. Dethlefsen L. Sargent M.. (2005). Diversity of the human intestinal Microbial flora. Science. 308, 1635–1638. doi: 10.1126/science.1110591 15831718 PMC1395357

[ref10] Fava F. Danese S. (2011). Intestinal microbiota in inflammatory bowel disease: friend of foe? World J. Gastroenterol. 17, 557–566. doi: 10.3748/wjg.v17.i5.557, PMID: 21350704 PMC3040327

[ref11] Feng R. Guo Z. K. Yan C. M. Li E. G. Tan R. X. Ge H. M. (2012). Anti-inflammatory flavonoids from Cryptocarya chingii. Phytochemistry. 76, 98–105. doi: 10.1016/j.phytochem.2012.01.007, PMID: 22277737

[ref12] Flannagan R. S. Brozyna J. R. Kumar B. Adolf L. A. Power J. J. Heilbronner S.. (2022). In vivo growth of staphylococcus lugdunensis is facilitated by the concerted function of heme and non-heme iron acquisition mechanisms. J. Biol. Chem 298:101823. doi: 10.1016/j.jbc.2022.101823, PMID: 35283192 PMC9052147

[ref13] Gilbert J. A. Field D. Swift P. Thomas S. Cummings D. Temperton B.. (2010). The taxonomic and functional diversity of microbes at a temperate coastal site: a 'multi-omic' study of seasonal and diel temporal variation. PLoS One 5:e15545. doi: 10.1371/journal.pone.0015545, PMID: 21124740 PMC2993967

[ref14] Girdhar S. R. Barta J. R. Santoyo F. A. Smith T. K. (2006). Dietary putrescine (1,4-diaminobutane) influences recovery of Turkey poults challenged with a mixed coccidial infection. J. Nutr. 136, 2319–2324. doi: 10.1093/jn/136.9.2319, PMID: 16920848

[ref15] He Q. Li S. Fan Y. Liu Y. Su Y. Zhou Z.. (2022). Complex Flavanones from Cryptocarya metcalfiana and structural revision of Oboflavanone A. J. Nat. Prod. 85, 1617–1625. doi: 10.1021/acs.jnatprod.2c00279, PMID: 35635020

[ref16] Hooper L. V. Gordon J. I. (2001). Commensal host-bacterial relationships in the gut. Science. 292, 1115–1118. doi: 10.1126/science.1058709, PMID: 11352068

[ref17] Hu G. Liu L. Miao X. Zhao Y. Li X. (2022). Research note: IsomiRs of chicken miR-146b-5p are activated upon salmonella enterica serovar Enteritidis infection. Poult. Sci. 101:101977. doi: 10.1016/j.psj.2022.101977, PMID: 35753206 PMC9249843

[ref18] Huang W. Zhang W. J. Cheng Y. Q. Jiang R. Wei W. Chen C. J.. (2014). Cytotoxic and antimicrobial flavonoids from Cryptocarya concinna. Planta Med. 80, 925–930. doi: 10.1055/s-0034-1368613, PMID: 25029174

[ref19] Julien L. A. Fau C. Baron F. Bonnassie S. Guérin-Dubiard C. Nau F.. (2020). The three Lipocalins of egg-white: only ex-FABP inhibits Siderophore-dependent iron sequestration by salmonella Enteritidis. Front. Microbiol 11:913. doi: 10.3389/fmicb.2020.00913, PMID: 32477312 PMC7242566

[ref20] Kaiser P. Rothwell L. Galyov E. E. Barrow P. A. Burnside J. Wigley P. (2000). Differential cytokine expression in avian cells in response to invasion by salmonella typhimurium, salmonella enteritidis and Salmonella gallinarum. Microbiology. 146, 3217–3226. doi: 10.1099/00221287-146-12-3217 11101679

[ref21] Lee S. La T. M. Lee H. J. Choi I. S. Song C. S. Park S. Y.. (2019). Characterization of microbial communities in the chicken oviduct and the origin of chicken embryo gut microbiota. Sci. Rep 9:6838. doi: 10.1038/s41598-019-43280-w, PMID: 31048728 PMC6497628

[ref22] Liu L. Lin L. Zheng L. Tang H. Fan X. Xue N.. (2018). Cecal microbiome profile altered by salmonella enterica, serovar Enteritidis inoculation in chicken. Gut pathogens 10:34. doi: 10.1186/s13099-018-0261-x, PMID: 30087697 PMC6074038

[ref23] Lopez C. A. Winter S. E. Rivera-Chávez F. Xavier M. N. Poon V. Nuccio S. P.. (2012). Phage-mediated acquisition of a type III secreted effector protein boosts growth of salmonella by nitrate respiration. MBio 3:e00143–e00112. doi: 10.1128/mBio.00143-12 22691391 PMC3374392

[ref24] Mon K. K. Saelao P. Halstead M. M. Chanthavixay G. Chang H. C. Garas L.. (2015). Salmonella enterica Serovars Enteritidis infection alters the indigenous microbiota diversity in young layer chicks. Front. Veterin. Sci 2:61. doi: 10.3389/fvets.2015.00061 PMC4672283 26664988

[ref25] Mukhopadhya I. Hansen R. El-Omar E. M. Hold G. L. (2012). IBD-what role do Proteobacteria play? Nat. Rev. Gastroenterol. Hepatol. 9, 219–230. doi: 10.1038/nrgastro.2012.14, PMID: 22349170

[ref26] Ochman H. Groisman E. A. (1996). Distribution of pathogenicity islands in salmonella spp. Infect. Immun. 64, 5410–5412. doi: 10.1128/iai.64.12.5410-5412.1996, PMID: 8945597 PMC174539

[ref27] Polansky O. Sekelova Z. Faldynova M. Sebkova A. Sisak F. Rychlik I. (2015). Important metabolic pathways and biological processes expressed by chicken Cecal microbiota. Appl. Environ. Microbiol. 82, 1569–1576. doi: 10.1128/AEM.03473-15, PMID: 26712550 PMC4771310

[ref28] Raffatellu M. George M. D. Akiyama Y. Hornsby M. J. Nuccio S. P. Paixao T. A.. (2009). Lipocalin-2 resistance confers an advantage to salmonella enterica serotype Typhimurium for growth and survival in the inflamed intestine. Cell Host Microbe. 5, 476–486. doi: 10.1016/j.chom.2009.03.011, PMID: 19454351 PMC2768556

[ref29] Thiennimitr P. Winter S. E. Winter M. G. Xavier M. N. Tolstikov V. Huseby D. L.. (2011). Intestinal inflammation allows salmonella to use ethanolamine to compete with the microbiota. Proc. Natl. Acad. Sci. U. S. A. 108, 17480–17485. doi: 10.1073/pnas.1107857108, PMID: 21969563 PMC3198331

[ref30] Videnska P. Sedlar K. Lukac M. Faldynova M. Gerzova L. Cejkova D.. (2014). Succession and replacement of bacterial populations in the caecum of egg laying hens over their whole life. PLoS One 9:e115142. doi: 10.1371/journal.pone.0115142, PMID: 25501990 PMC4264878

[ref31] Winter S. E. Thiennimitr P. Winter M. G. Butler B. P. Huseby D. L. Crawford R. W.. (2010). Gut inflammation provides a respiratory electron acceptor for salmonella. Nature. 467, 426–429. doi: 10.1038/nature09415, PMID: 20864996 PMC2946174

