# Supplementary figures and images for: The response of cecal microbiota to inflammatory state induced by Salmonella enterica serovar Enteritidis
Source: Front Microbiol. 2022 Aug 25;13:963678. doi: 10.3389/fmicb.2022.963678 (PMC9453680; doi:10.3389/fmicb.2022.963678)

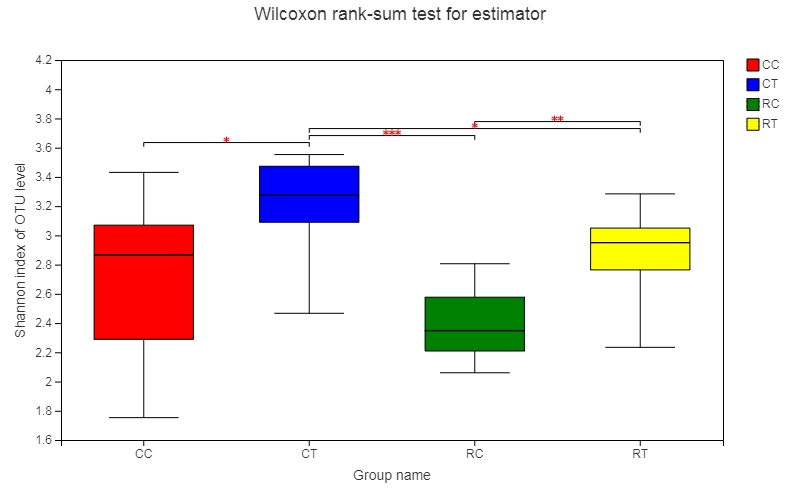

Supplement: SUPPLEMENTARY FIGURE S1 — Shannon index of CC, CT, RC, and RT. [file Image_1.JPEG]

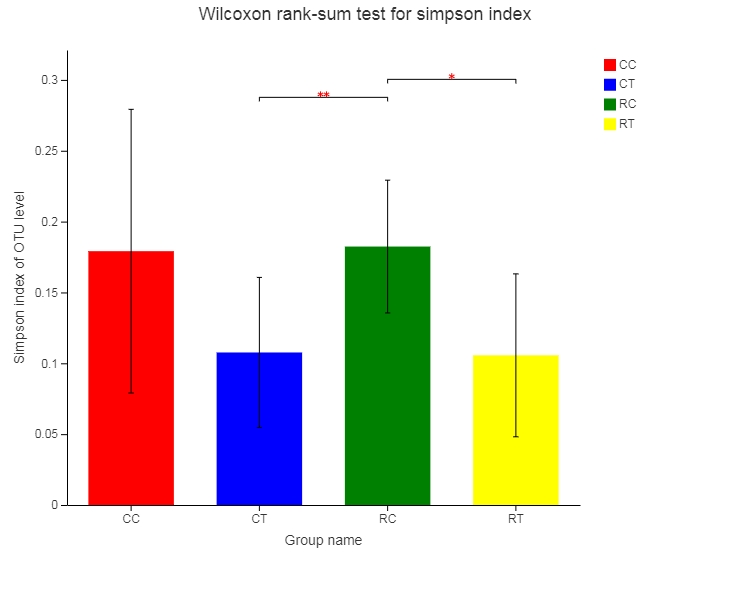

Supplement: SUPPLEMENTARY FIGURE S2 — Simpson index of CC, CT, RC, and RT. [file Image_2.JPEG]
